# Supplementary material for: Pre-Existing Tumoral B Cell Infiltration and Impaired Genome Maintenance Correlate with Response to Chemoradiotherapy in Locally Advanced Rectal Cancer
Source: Cancers (Basel). 2020 Aug 10;12(8):2227. doi: 10.3390/cancers12082227 (PMC7464257; doi:10.3390/cancers12082227)
Supplement: Supplementary file 1 [file cancers-12-02227-s001.zip › Supp_Figures_Cancers_2020518.pdf]

Supplementary Figure S1

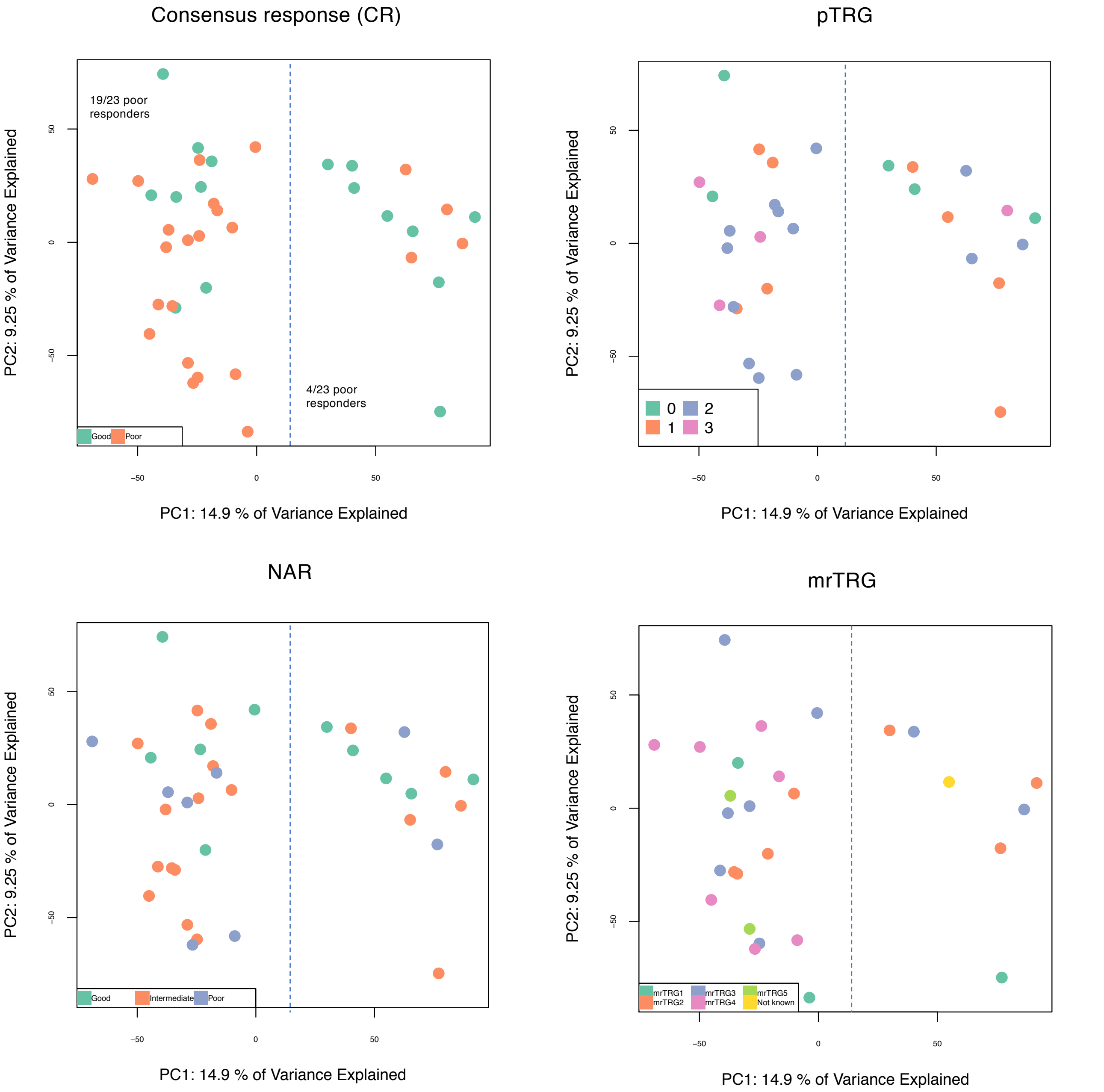

Supplementary Figure S1. Principal component analysis (PCA) from global gene expression data.
Principal component analysis (PCA) resulting from all gene expression data, where each dot represents a patient. The plot was labeled according to response assessment variables to establish the most plausible dichotomic driver of the observed spatial distribution: **(a)**: Consensus Response (CR) classification; **(b)**: Pathological tumor regression (pTRG) score; **(c)**: Neoadjuvant rectal (NAR) score; **(d):** Response assessment by MRI (mrTRG). Some of the plots contain “missing” dots corresponding to missing data (e.g., irresectable patients were classified as poor responders in our Consensus Response classification, but their pathological score was not available).

Supplementary Figure S2

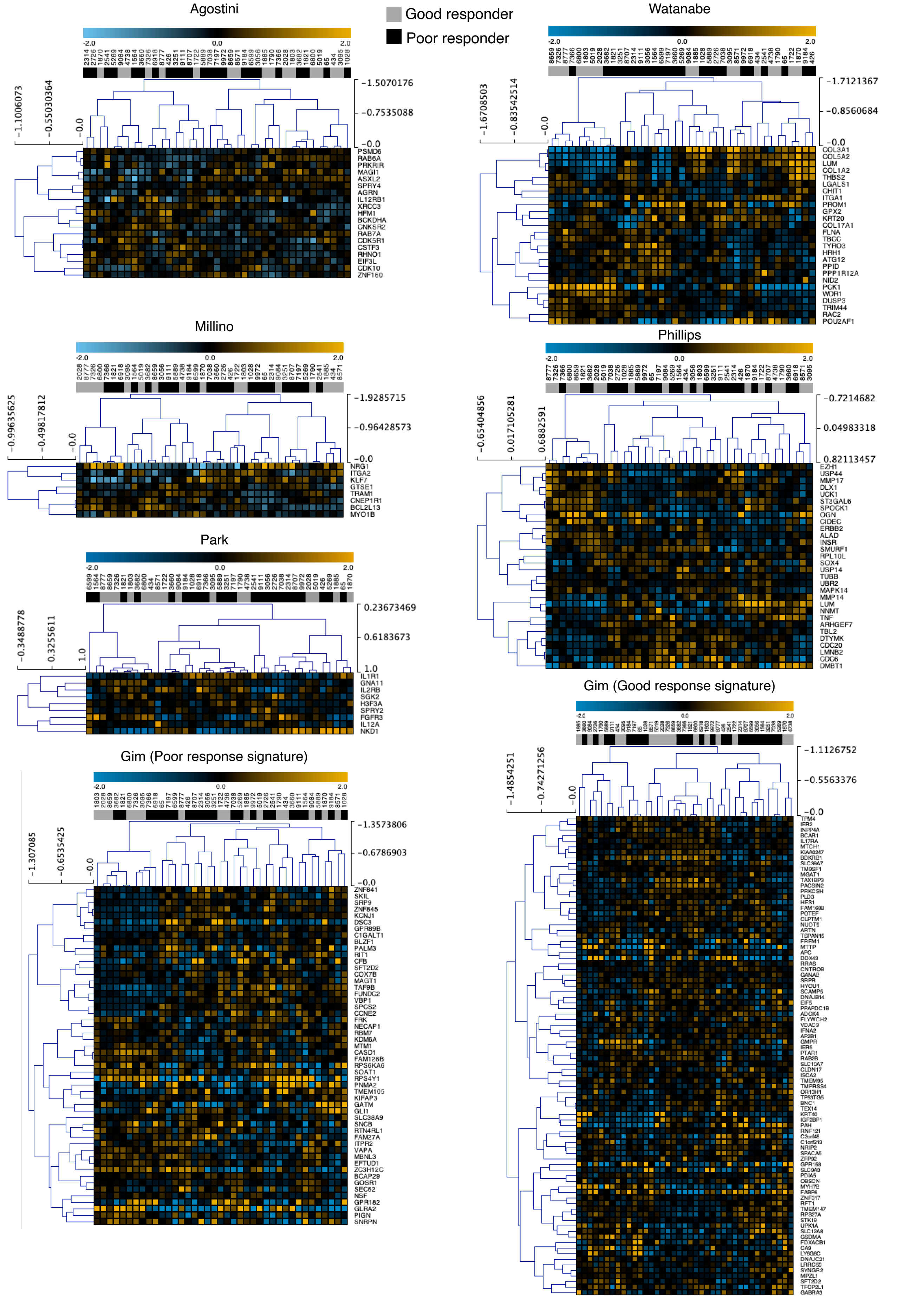

Supplementary Figure S2. Performance of previous external predictive signatures in our cohort. Heatmaps showing unsupervised clustering of our cohort patients based on different published predictive gene expression signatures (First author's name on top of each heatmap). None of these previously proposed signatures are able to group our cohort's good and poor responders robustly.

Supplementary Figure S3

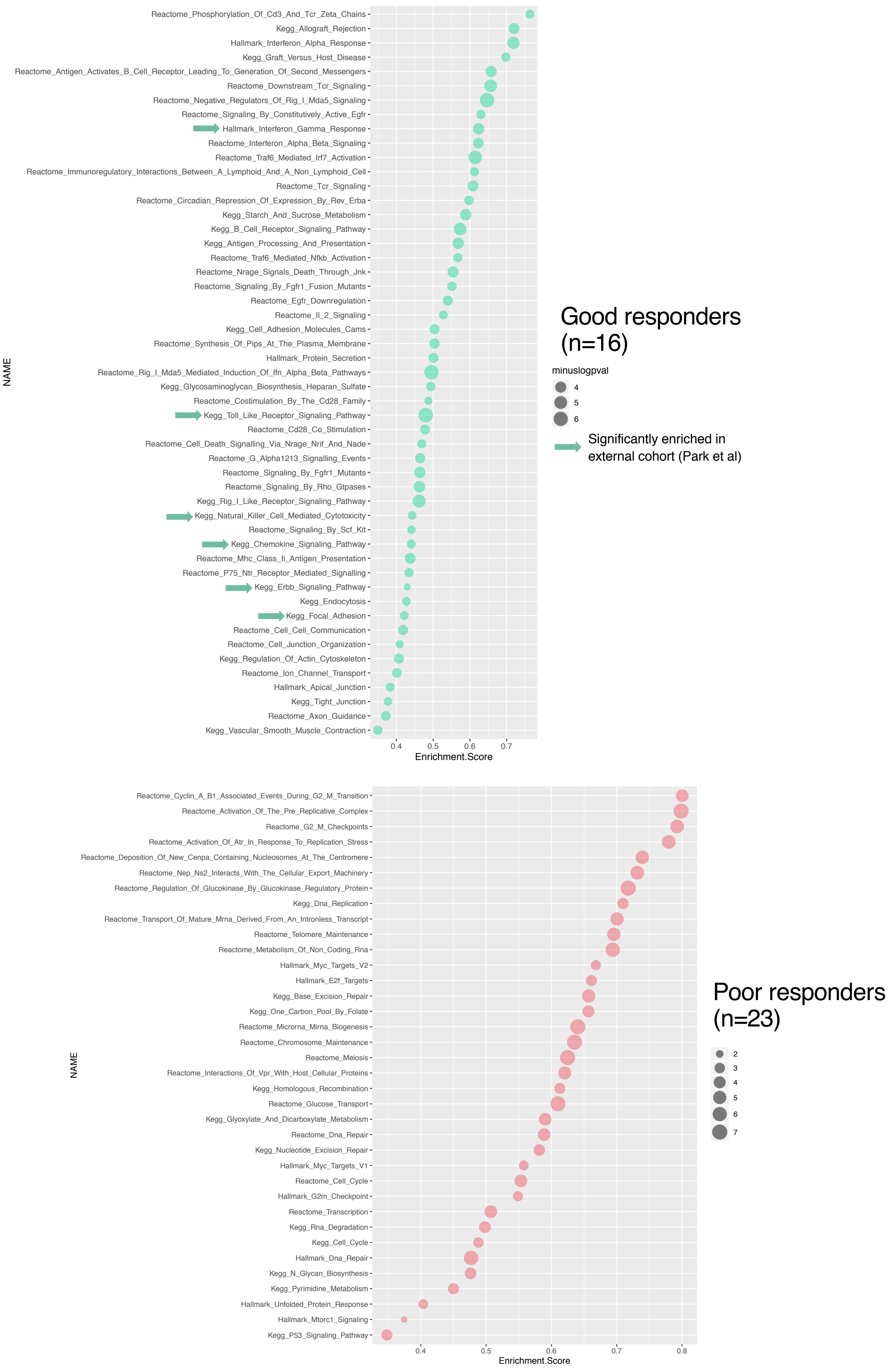

Supplementary Figure S3. Significantly enriched terms in GSEA-P analysis.
Bubble plots displaying significantly enriched cellular processes ($p<0.05$) in good responders (green) and poor responders (red) obtained by GSEA-P. The y-axis shows the name of each gene set, while the x-axis corresponds to GSEA enrichment scores. The size of each bubble is proportional to each geneset's negative log(p-value), therefore a bubble size larger than 1.3 corresponds to $p<0.05$. Green arrows indicate genesets also significantly enriched in the largest external cohort available to date, (Park et al), coincident with enhanced immune features in good responders.

Supplementary Figure S4

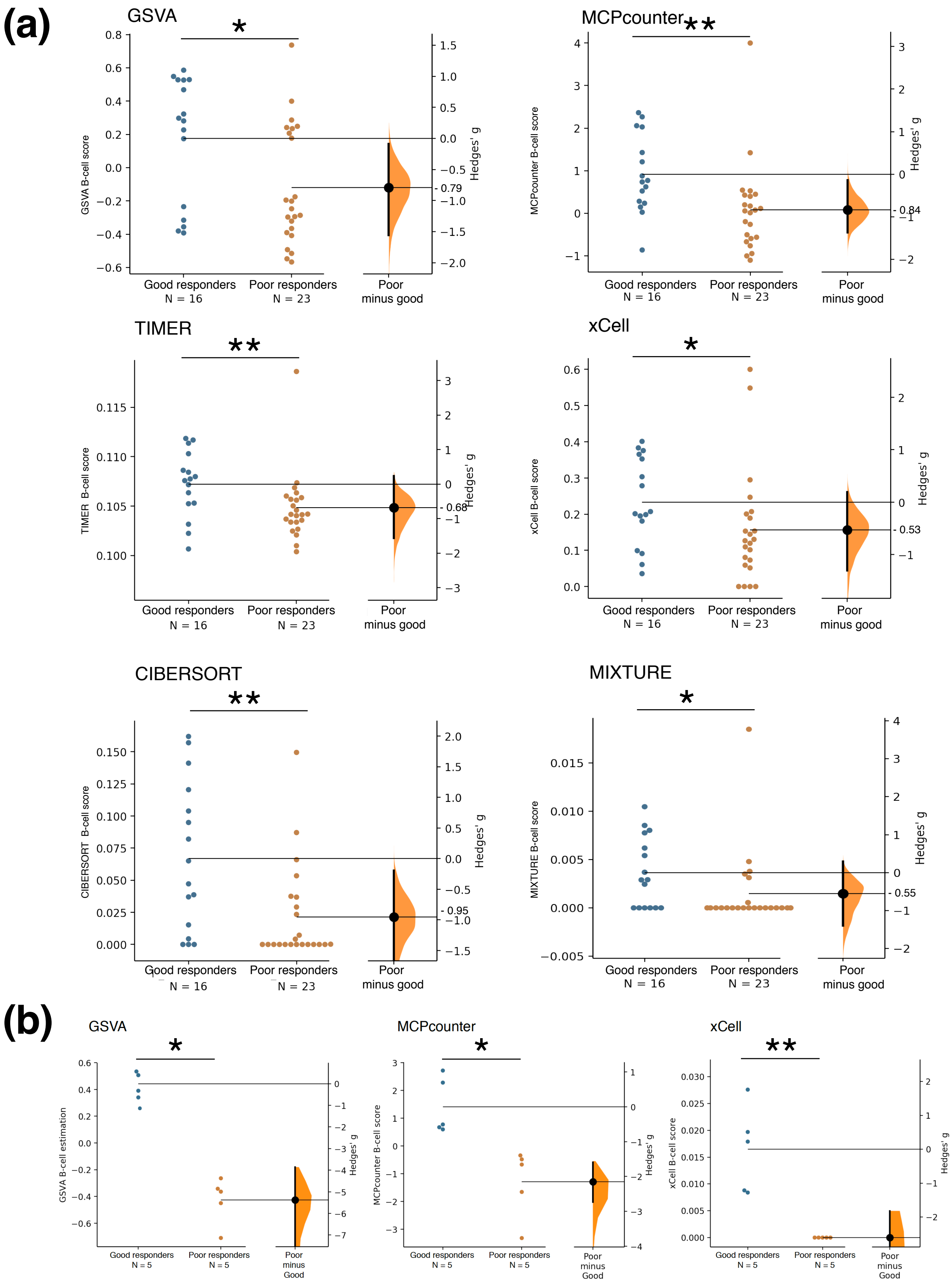

Supplementary Figure S4. B cell estimations using deconvolution algorithms.
The Hedges' g between good and poor responders is shown in the Gardner-Altman estimation plots based on tumor biopsy B cell proportion data obtained by different in silico estimators, using two technologies: microarrays (**a**, n=39) and RNAseq (**b**, n=10). For each plot, both patient groups are plotted on the left axes; the mean difference is plotted on floating axes on the right as a bootstrap sampling distribution. The mean difference is depicted as a dot; the 95% confidence interval is indicated by the ends of the vertical error bar.

Supplementary Figure S5

(a)

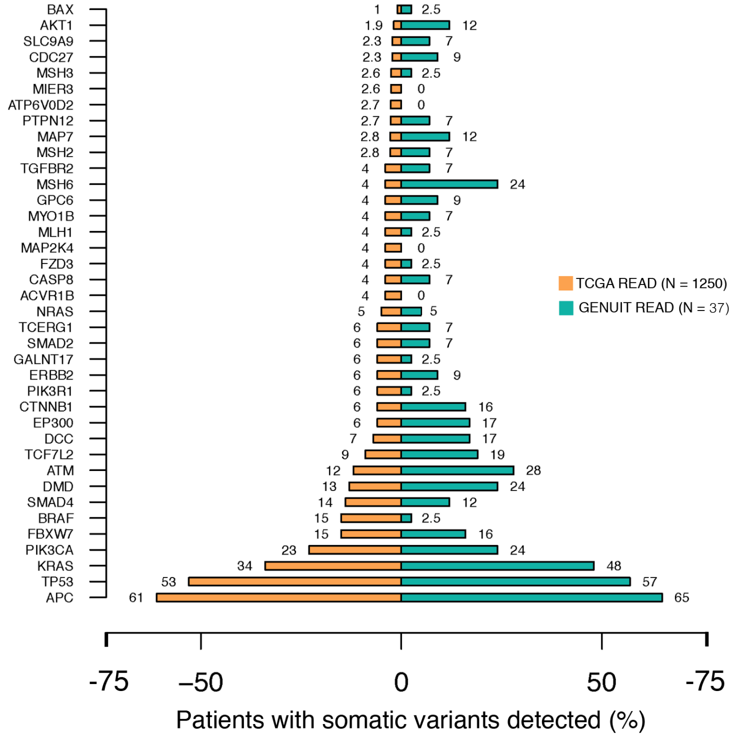

(b)

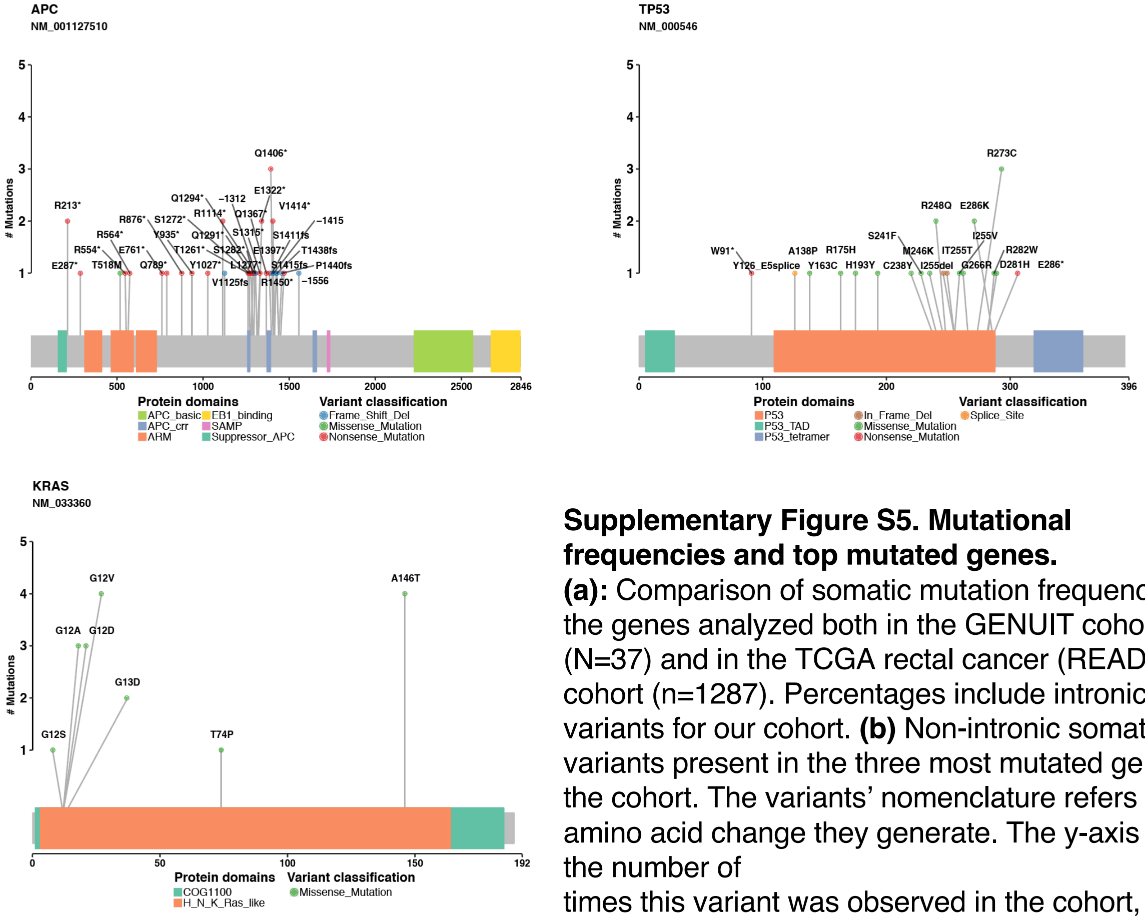

Supplementary Figure S5. Mutational frequencies and top mutated genes.
(a): Comparison of somatic mutation frequencies in the genes analyzed both in the GENUIT cohort (N=37) and in the TCGA rectal cancer (READ) cohort (n=1287). Percentages include intronic variants for our cohort. **(b)** Non-intronic somatic variants present in the three most mutated genes in the cohort. The variants' nomenclature refers to the amino acid change they generate. The y-axis shows the number of

times this variant was observed in the cohort, while the x-axis marks the position of the amino acid they affect. Color references indicate protein domains and variant classification. For *APC*, *TP53* and *KRAS* genes, the presence of mutational hotspots containing high impact variants is clearly observed.

Supplementary Figure S6

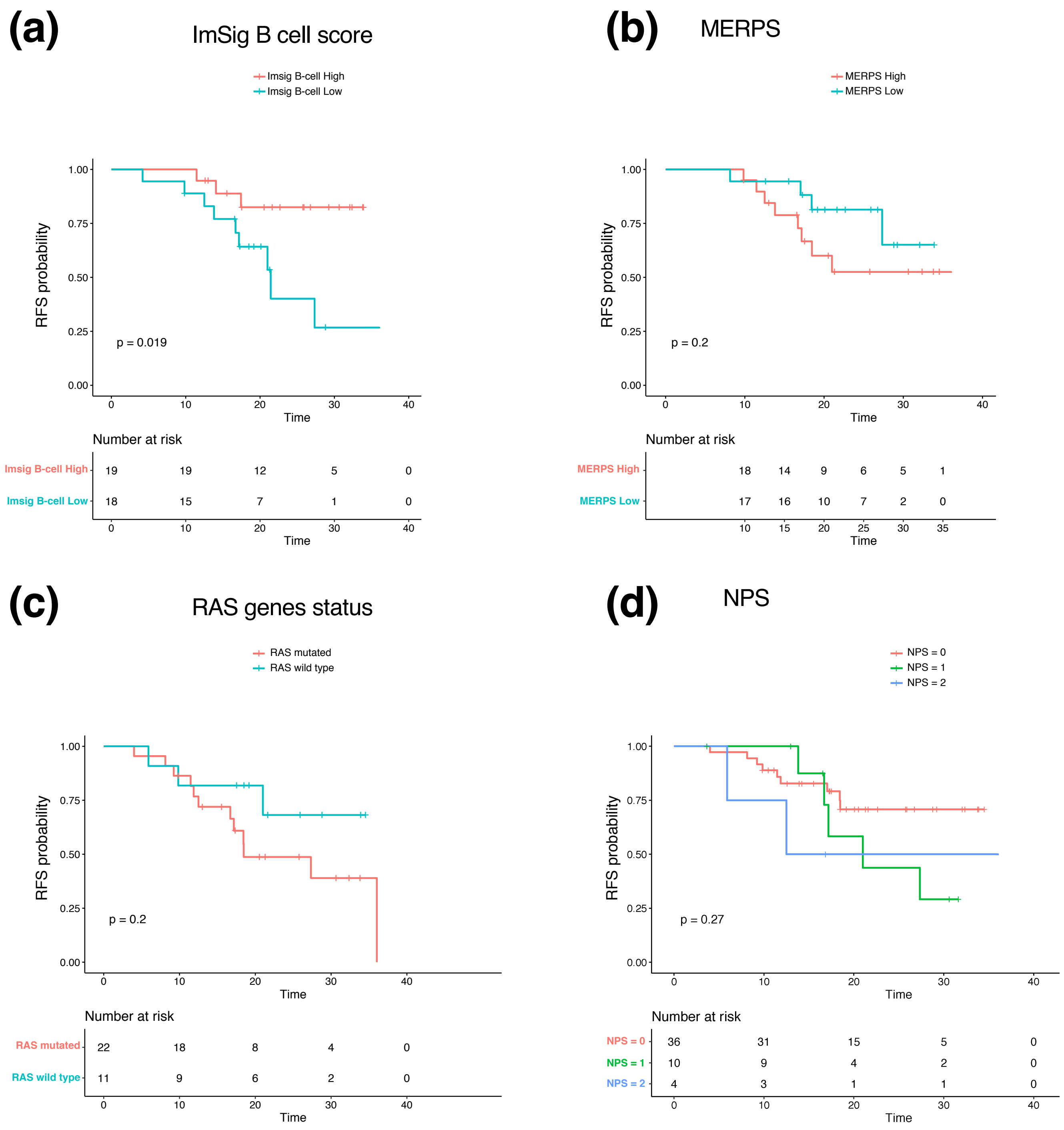

Supplementary Figure S6. Prognostic value of molecular predictive variables. Kaplan-Meier analyses evaluating the prognostic value of significant molecular predictive variables for recurrence-free survival (RFS). **(a)** Median split B cell score derived from ImSig; **(b)** Median repair pathway score (MERPS), a score we derived from PARADIGM analysis; **(c)** Mutational status of *RAS* genes; **(d)** Neutrophil-platelet score (NPS).
